# Bee sting envenomation severe cases in Manaus, Brazilian Amazon: clinical characteristics and immune markers of case reports

**DOI:** 10.1590/0037-8682-0319-2021

**Published:** 2020-12-21

**Authors:** Iran Mendonça-da-Silva, Wuelton Marcelo Monteiro, Jacqueline Almeida Gonçalves Sachett, Endila Souza Barbosa, Marcelo Cordeiro-dos-Santos, Marcus Vinícius Guimarães Lacerda, Gisely Cardoso Melo, Allyson Guimarães Costa, Fernando Fonseca Almeida Val

**Affiliations:** 1 Universidade do Estado do Amazonas, Programa de Pós-Graduação em Medicina Tropical, Manaus, AM, Brasil.; 2 Fundação de Medicina Tropical Dr. Heitor Vieira Dourado, Instituto de Pesquisa Clínica Carlos Borborema, Manaus, AM, Brasil.; 3 Fundação Alfredo da Matta, Diretoria de Ensino e Pesquisa, Manaus, AM, Brasil.; 4 Instituto Leônidas & Maria Deane, Manaus, AM, Brasil.; 5 Universidade Federal do Amazonas, Instituto de Ciências Biológicas, Programa de Pós-Graduação em Imunologia Básica e Aplicada, Manaus, AM, Brasil.; 6 Fundação Hospitalar de Hematologia e Hemoterapia do Amazonas, Diretoria de Ensino e Pesquisa, Manaus, AM, Brasil.

**Keywords:** Africanized bees, Inflammatory response, Systemic injury

## Abstract

Bee venom is a natural toxin composed of several peptides. Massive envenoming causes severe local and systemic reactions. We report two cases of severe bee envenomation, of which one was fatal. We also describe clinical characteristics and immune markers. Both victims suffered from respiratory distress, renal failure, rhabdomyolysis, and shock. They required invasive mechanical ventilation, vasoactive drugs, and renal replacement therapy. Moreover, serum levels of chemokines, cytokines, and cell-free circulating nucleic acids demonstrated an intense inflammatory process. Massive envenoming produced systemic injury in the victims, with an uncontrolled inflammatory response, and a more significant chemotactic response in the fatal case.

## INTRODUCTION

In 1956, African bees were imported to Brazil to crossbreed with European bees for commercial purposes. Accidentally, African queens initiated the Africanization of other species of honeybees in the Americas. Africanized bees are aggressive and can attack in large groups. This may result in the death of a patient due to the high volume of venom inoculation^1^. In Brazil, between 2000 and 2018, overall, 159,520 cases of bee stings were reported to the official surveillance system. These resulted in 466 deaths (0.3%). In the same period, the Brazilian Amazon recorded 6,799 cases and 22 deaths, primarily in urban areas, with similar lethality^2^. 

Bee venom (BeV) is a natural toxin comprising peptides, including melittin, adrenaline, dopamine, histamine, hyaluronidase, noradrenaline, phospholipases A_2_ and B, and serotonin, apamin, and melittin^3^. After a massive envenomation by Africanized bees, clinical features include intravascular hemolysis, bleeding, acute respiratory distress syndrome, hypertension, myocardial damage, hepatic dysfunction, acute renal failure (ARF), rhabdomyolysis, shock, and coma^3^.

The components of BeV have several local and systemic actions^4^. Immunological interactions between the venom and immune cells show a pattern of response mediated by mast cells, which are activated by immunoglobulin E (IgE) antibodies. BeV components may induce faster systemic envenomation through hyaluronidase (spreading factor) and hydrolyzed hyaluronan fragments due to its pro-inflammatory, pro-angiogenic, and immunostimulatory properties, thus leading to rapid spread and massive envenoming^5^. 

The inflammatory processes and clinical characteristics involved in massive envenoming that evolve to death remain poorly understood. In this study, we report two cases of severe bee stings that were treated at the *Fundação de Medicina Tropical Doutor Heitor Vieira Dourado* (FMT-HVD), a tertiary hospital in Manaus, Western Brazilian Amazon. This study was approved by the local Ethical Review Board (process #713.140).

## CASE REPORTS

**Case 1:** A 70-year-old man, who resided in a rural area of Careiro da Várzea (approximately 30 km from Manaus by river) presented to the FMT-HVD emergency department (ED), with a sore throat, myalgia, nausea, three vomiting episodes, diarrhea, anuria, and breathlessness. The patient was reported to have been stung by a swarm of bees while working in a rural area. Mild jaundice (+/4+) was noted. Local signs and symptoms included pain, edema, and pruritus in the affected area. The patient was diagnosed with uncontrolled diabetes and hypertension and was treated with enalapril. No allergies were reported.

**FIGURE 1:**
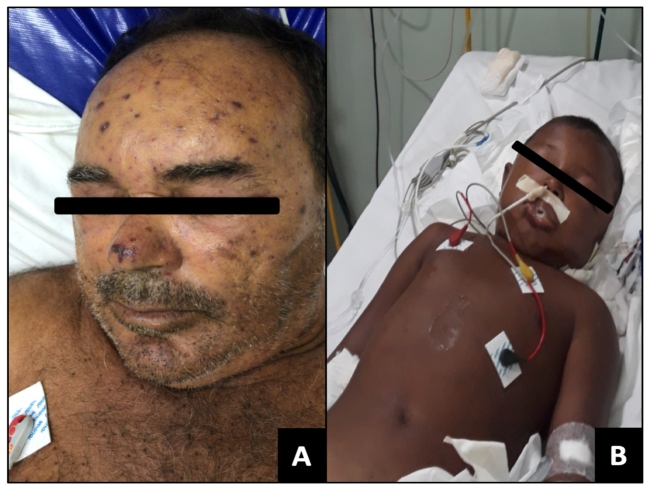
Clinical presentation of the patients. **1(A):** Case 1 on admission; photo shows bee stings to the head. **1(B):** Case 2 intubated in pediatric ICU; Patient presented tachypnea, swollen face, eyelids and lip edema, myalgia, and hardened edema in his chest.

Physical examination revealed massive bee stings to the head, thorax, arms and legs (Figure 1A), hypertension (blood pressure [BP] = 200/100 mmHg), tachycardia (heart rate [HR]=118 bpm), dyspnea, and hyperglycemia (329 mg/dL), although no fever. The time between being stung and hospital admission was ~20 h. Respiratory failure was treated with adrenaline, dexamethasone, methylprednisolone, fenoterol, hydrocortisone, and dexchlorpheniramine. Subsequently, furosemide and captopril were administered. The remaining embedded stings were removed. Despite a slight clinical improvement, the patient developed agitation, dysphonia, apathy, hyperglycemia (313 mg/dL), BP elevation (189/111 mmHg), tachycardia (120 bpm), and tachypnea (respiratory rate [RR] = 35 breaths/min). The patient was transferred to the intensive care unit (ICU). The patient was dehydrated, scored 13 on the Glasgow coma scale, and RR was stabilized with non-invasive oxygen supplementation. However, no electrocardiographic alterations were identified, and upper respiratory tract swelling was absent. 

Supplementary material: Table 1 presents the laboratory parameters obtained in Case 1. Six hours after admission, the patient presented with swelling in his throat, laryngeal stridor, respiratory distress, and profuse sweating. The patient was not responsive to epinephrine or hydrocortisone nebulization. The patient developed peripheral cyanosis and hypotension, which was treated with norepinephrine. Endotracheal intubation was performed. The tongue, glottis, and epiglottis were swollen. During intubation, the patient developed seizures, which stopped after mechanical ventilation. Peripheral cyanosis and anuria persisted 8 h after intubation. Cardiorespiratory arrest and death occurred after resuscitation attempts. A necropsy was not authorized by the family.

**Case 2:** A 10-year-old boy, residing in the rural area of Urucurituba, approximately 339 km from Manaus by river, presented to the FMT-HVD in a somnolent state, but conscious, after suffering a massive bee attack the previous day while playing. Physical examination revealed ~200 bee stings to the head, face, neck, upper limbs, and trunk. No comorbidities were reported. Time between the stinging and hospital admission was 24 h. Intense pain, edema, and pruritus in the affected areas were reported. 

**FIGURE 2:**
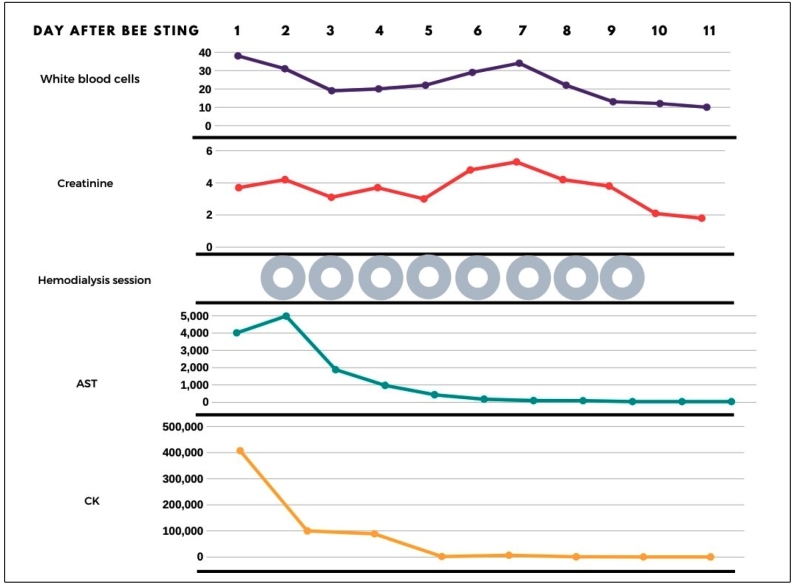
Follow-up of Case 2. Rhabdomyolysis is a well-known cause of acute kidney injury and renal failure. Details of laboratory tests are presented in Supplementary material: Table 2.

On physical examination, the patient presented with tachypnea, a swollen face, eyelids and lip edema, mild jaundice (+/4+), myalgia, sialorrhea, moaning, hardened chest edema, and a flat rigid abdomen painful to palpation, diarrhea, and elevated serum creatinine (Figures 1B and Figure 2). The remaining stingers were removed. The patient was transferred to the pediatric ICU and intubated. Adrenaline, methylprednisolone, fenoterol, hydrocortisone, and dexchlorpheniramine were administered. Two days after admission, elevated muscle enzymes, aminotransferases, and lactate dehydrogenase revealed severe rhabdomyolysis (Figure 2). Oliguric ARF required eight hemodialysis sessions. 

Supplementary material: Table 2 presents the detailed laboratory follow-up of Case 2. The patient developed a hypertensive crisis, which was treated with captopril, amlodipine, and losartan. Abdominal ultrasonography revealed mild ascites, alithiasic cholecystitis, splenomegaly, and bilateral acute glomerulopathy. Otorrhea, with neutrophilia and fever, was treated with ceftriaxone, metronidazole, and clindamycin. The patient improved, and was discharged after 15 days. No pain or discomfort was reported during follow-up.

**FIGURE 3:**
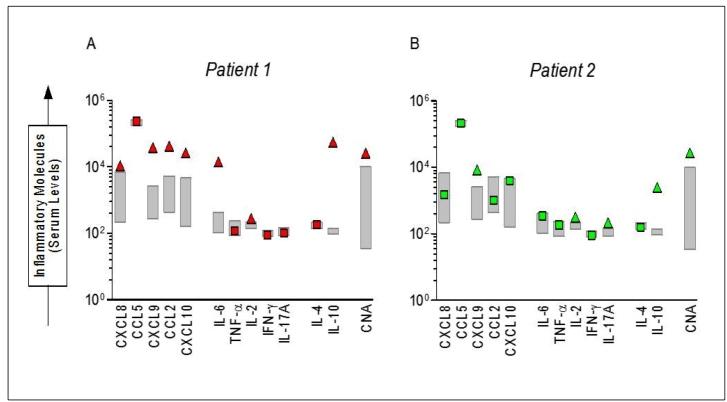
Serum levels of chemokines, cytokines and cell-free nucleic acids in severe bee sting cases at hospital admission. 3(A): Case 1. 3(B): Case 2. The gray boxes represent serum levels of a control group, that consisted of 20 healthy subjects, ages ranging from 22 to 36 years (median = 27 years).

**Circulating immune marker level measurements in both cases:** Levels of circulating chemokines, cytokines, and nucleic acids (CNAs) are shown in Figure 3 for both cases. Supplementary material: Material and Methods shows how the measurements of circulating immune markers were performed. There was an increase in the levels of chemokines (CXCL-8, CXCL-9, CCL-2, and CXCL10) in Case 1, considering the 95% CI of the mean values observed for a control group of age-matched healthy individuals. However, only the CXCL-9 chemokine was increased in Case 2. No difference was observed in CCL-5. The pro-inflammatory cytokine IL-6 level increased in Case 1. The regulatory cytokine IL-10, cell proliferation cytokine IL-2, and CNAs increased in both patients.

## DISCUSSION

Bee stings cause pain, edema, nausea, pruritus, erythema, vomiting, diarrhea, myalgia, respiratory distress, tachycardia, and vomiting, as previously reported^3^. Moreover, both cases presented exuberant clinical manifestations due to massive bee stings and the volume of venom injected. In fact, both cases showed signs of respiratory distress, intense rhabdomyolysis, and increased levels of inflammatory markers. Furthermore, the dynamic river topography in the Amazon greatly delays access to proper health care.

Multiple bee stings always constitute a medical emergency, and patients who have suffered a mass attack by Africanized bees (>50 stings) should be monitored, as the venom may persist in circulation for days and cause delayed reactions^2^. Inflammation results from an unbalanced immune response associated with the elevated production of pro-inflammatory cytokines, chemokines, and histamine. These are secreted through the degranulation of mast cells triggered by allergen stimuli, which are central in acute hypersensitivity reactions. This hypersensitivity inflammation has been associated with mitogen-activated protein kinase and nuclear factor-κB signaling cascade of mast cells and leukocytes with an inhibitory effect. This effect has been shown to be dependent on BeV concentrations^6^.

The immune profile of Case 1 showed a remarkable increase in chemokines, which was probably associated with severe envenomation owing to the massive envenoming, as the patient did not mention any bee allergy, excluding anaphylaxis. The consequences of an increase in immune-mediated reactions secondary to massive exposure to venom might have produced acute envenoming syndrome and immune regulation^1^ ^,^ ^3^. Moreover, BeV action is dependent on the time, dose, and type of cell affected, producing a significant suppression of the immune response, with less leukocyte migration and concentrations of inflammatory mediators^5^. Hyperglycemia can also be considered a risk factor during envenoming. This has been shown to be associated with cytokine-mediated disruption of insulin secretion in severe scorpion envenoming. 

The presence of comorbidities in Case 1 may have influenced the higher levels of pro-inflammatory biomarkers and, consequently the patient’s outcome. The exact extent to which both comorbidities contribute to immune regulatory exhaustion and clinical outcomes in such cases is currently unknown. Unfortunately, venom-specific IgE level analysis to confirm hypersensitivity to BeV allergens and plasma mast cell tryptase activity were not available for either patient. However, it is possible that the wide inflammatory activation with extensive release of anaphylactogenic mediators may have contributed to anaphylactic symptoms and clinical outcomes (bronchoconstriction, hypotension, angioedema, abdominal pain, etc.).

Envenoming, such as those generated by BeV, induces an intense inflammatory process, in which neutrophils influence the concentrations of other inflammatory molecules. Although we did not perform absolute counts of neutrophils, eosinophils, basophils, lymphocytes, and monocytes, Case 1 presented a marked increase in chemokines (CXCL-8, CXCL-9, CCL-2, and CXCL10). The high levels of these chemokines released by endothelial cells in the skin and mucosal bite locations, promoted intense cell recruitment and inflammatory response^7^. In many cases, chemokines activate target cells, increasing the production of inflammatory molecules, oxygen radicals, and nitric oxide, which induce the degranulation of neutrophils and other cell types. Furthermore, this response contributes to host immune defense locally. However, the exacerbated production of these inflammatory molecules promotes tissue destruction and impaired function of the affected organs^7^.

Regarding toxic reactions, rhabdomyolysis was the hallmark of both cases, and both evolved to renal failure. One possible mechanism for muscle damage involves the direct action of venom toxic components, such as melittin and phospholipase A2, on skeletal muscle tissue^8^. Generally, rhabdomyolysis causes problems that range from asymptomatic elevations of creatine kinase (CK) to life-threatening electrolyte changes and severe acute kidney injury. An in-hospital mortality risk score for patients with rhabdomyolysis may be obtained using demographic, clinical, and laboratory admission data. According to this score, both patients presented a 60% risk of death and a need for continuous renal replacement therapy^9^. 

In this study, an elevation of CK-MB levels was recorded in both patients. Enzymatic changes and morphological lesions of the acute myocardial infarction type were found in rats, showing a possible direct toxic action of the venom on cardiac muscle^1^. The severe toxic effects of BeV would ideally be treated with antivenom. Although there have been several successful attempts to produce bee antivenoms, there are no antivenoms currently available in Brazil^10^. 

Increased levels of CNAs have been previously reported in sepsis, trauma, and cancer. Furthermore, they are speculated to be useful biomarkers for clinical outcomes. Their origin is debatable. CNAs have been associated with neutrophil extracellular traps^11^. Nonetheless, an experimental sepsis model showed otherwise, which led us to hypothesize an association with necrotic cells^11^. Besides, the association between BeV and DNA damage is controversial^12^. In this report, both cases presented higher CNAs than controls. However, neutrophils were not quantified. The relationship between CNAs and neutrophils was not established, and therefore deserves further investigation in massive bee envenoming.

According to the two cases presented here, the patient that evolved to death presented a clinical case of severe respiratory distress and more significant inflammatory/chemotactic response when compared to the patient with predominant rhabdomyolysis and milder respiratory distress, despite being under intensive clinical support. Thromboembolic and hemorrhagic markers were not available for these patients. Nonetheless, such markers would help to further understand the pathogenesis and clinical outcomes. Immune markers, such as chemokines (CXCL-8, CXCL-9, CCL-2, and CXCL10), also seem to be distinct between the two clinical presentations, which could be used as predictors of severity. This study depicts the outcome-impacting aspects of bee sting envenomation in the Amazon: delayed access to proper health care, the complex management of such cases, and how intricate the immune response is to massive loads of BeV, besides how this appears to influence the clinical outcome. Prospective investigation of the BeV immune response is warranted to further comprehend such aspects.

## ETHICS APPROVAL

This study was approved by the local Ethics Review Board (Approval #713.140). The family gave consent in both cases.

## References

[1] Ferreira RS, Almeida RAMB, Barraviera SRCS, Barraviera B (2012). Historical Perspective and Human Consequences of Africanized Bee Stings in the Americas. J Toxicol Environ Health B Crit Rev.

[2] Ministério da Saúde (MS). Secretaria de Vigilância em Saúde. Sistema Nacional de Vigilância em Saúde (2019). Acidentes de trabalho por animais peçonhentos entre trabalhadores do campo, floresta e águas, Brasil 2007 a 2017.

[3] Pucca MB, Cerni FA, Oliveira IS, Jenkins TP, Argemí L, Sørensen CV (2019). Bee Updated: Current Knowledge on Bee Venom and Bee Envenoming Therapy. Front Immunol.

[4] Moon D-O, Park S-Y, Lee K-J, Heo M-S, Kim K-C, Kim M-O (2007). Bee venom and melittin reduce proinflammatory mediators in lipopolysaccharide-stimulated BV2 microglia. Int Immunopharmacol.

[5] Komi DEA, Shafaghat F, Zwiener RD (2018). Immunology of Bee Venom. Clin Rev Allergy Immunol.

[6] Kang YM, Chung KS, Kook IH, Kook YB, Bae H, Lee M (2018). Inhibitory effects of bee venom on mast cell-mediated allergic inflammatory responses. Int J Mol Med.

[7] Garcia-Ramallo E, Marques T, Prats N, Beleta J, Kunkel SL, Godessart N (2002). Resident cell chemokine expression serves as the major mechanism for leukocyte recruitment during local inflammation. J Immunol.

[8] Ownby CL, Powell JR, Jiang M, Fletcher JE (1997). Melittin and phospholipase A2 from bee (Apis mellifera) venom cause necrosis of murine skeletal muscle in vivo. Toxicon.

[9] McMahon GM, Zeng X, Waikar SS (2013). A risk prediction score for kidney failure or mortality in rhabdomyolysis. JAMA Intern Med.

[10] Barbosa AN, Boyer L, Chippaux J-P, Medolago NB, Caramori CA, Paixão AG (2017). A clinical trial protocol to treat massive Africanized honeybee (Apis mellifera) attack with a new apilic antivenom. J Venom Anim Toxins Incl Trop Dis.

[11] Margraf S, Lögters T, Reipen J, Altrichter J, Scholz M, Windolf J (2008). Neutrophil-derived Circulating Free DNA (cf-DNA/NETs): A Potential Prognostic Marker for Posttraumatic Development of Inflammatory Second Hit and Sepsis. Shock.

[12] Gajski G, Domijan AM, Garaj-Vrhovac V (2012). Alterations of GSH and MDA Levels and Their Association With Bee Venom-Induced DNA Damage in Human Peripheral Blood Leukocytes. Environ Mol Mutagen.

