# Supplementary material for: Bee sting envenomation severe cases in Manaus, Brazilian Amazon: clinical characteristics and immune markers of case reports
Source: Rev Soc Bras Med Trop. 2020 Dec 21;54:e20200319. doi: 10.1590/0037-8682-0319-2021 (PMC7747811; doi:10.1590/0037-8682-0319-2021)
Supplement: Supplementary file 1 [file 1678-9849-rsbmt-54-e20200319-suppl1.pdf]

**Supplementary material: TABLE 1:** Laboratory parameters obtained from Case 1.

| Parameters | April 25th, 2019 | April 26th, 2019 |
|---|---|---|
| Hemoglobin (g/dL) | 15.5 | 15.7 |
| White blood cells ($10^3$/$mm^3$) | 27.9 | 38.4 |
| Platelets ($10^3$/$mm^3$) | 438.9 | 328.6 |
| Creatinine (mg/dL) | 4.1 | 7.1 |
| Alkaline phosphatase (IU/L) | 251 | 340 |
| Gamma-glutamyl transferase (IU/L) | 26 | 36 |
| Total bilirubin (mg/dL) | 1.33 | 1.23 |
| Direct bilirubin (mg/dL) | 0.02 | 0.02 |
| Indirect bilirubin (mg/dL) | 1.31 | 1.21 |
| Glucose (mg/dL) | 344 | 149 |
| Potassium (mmol/L) | 6.9 | 7.9 |
| Sodium (mmol/L) | 135 | 137 |
| Aspartate aminotransferase (IU/L) | 513 | 1998 |
| Alanine aminotransferase (IU/L) | 62 | 384 |
| Urea (mg/dL) | 99 | 174 |
| Albumin (g/dL) | 4.2 | 3.5 |
| Creatine phosphokinase (IU/L) | 19,995 | 40,801 |
| Creatine phosphokinase-MB (IU/L) | 381 | 606 |
| Lactate dehydrogenase (IU/L) | 1036 | 8604 |
| pH | 7.41 | 7.07 |
| $pCO_2$ | 22.1 | 42.9 |
| $pO_2$ | 69.3 | 50.3 |
| $sO_2$ | 93.1 | 74.6 |
| Lactate (mg/dL) | 44 | 69 |
| Base excess (mmol/L) | -9.8 | -16.3 |
| $HCO_3$- (mmol/L) | 13.9 | 11.9 |

**Reference values:** Hemoglobin: 13.0-16.0 g/dL; White blood cells: 4.000-10.000/mm³; Platelets: 130,000-400.000/mm³; Creatinine: 0.5-1.2 mg/dL for adults; Alkaline phosphatase: male ≤390 IU/L; Gamma-glutamyl transferase: 7-60 IU/L for males; Total bilirubin: 0.01-1.3 mg/dL; Direct bilirubin: 0.01-0.3 mg/dL; Indirect bilirubin: 0.01-0.7 mg/dL; Glucose: <99 mg/dL; Potassium: 3.6-5.2 mmol/L; Sodium: 135-145 mmol/L; Aspartate aminotransferase: 2-38 IU/L; Alanine aminotransferase: 2-44 IU/L; Urea: 10-45 mg/dL; Albumin: 3.5-5 g/dL; Creatine phosphokinase: 24-190 IU/L; Creatine phosphokinase-MB: 2-25 IU/L; Lactate dehydrogenase: 211-423 IU/L; pH: 7.35-7.45; $pCO_2$: 35-45; $pO_2$: 80-100; $sO_2$: 94-97%; Lactate ≥45 mg/dL; Base excess -2 to +2 (mmol/L); $HCO_3^-$: 22-26 mmol/L.
