# Supplementary material for: Bee sting envenomation severe cases in Manaus, Brazilian Amazon: clinical characteristics and immune markers of case reports
Source: Rev Soc Bras Med Trop. 2020 Dec 21;54:e20200319. doi: 10.1590/0037-8682-0319-2021 (PMC7747811; doi:10.1590/0037-8682-0319-2021)
Supplement: Supplementary file 2 [file 1678-9849-rsbmt-54-e20200319-suppl2.pdf]

Supplementary material: TABLE 2: Laboratory parameters obtained from Case 2.

**Parameters**	**July 18th, 2019**	**July 19th, 2019**	**July 20th, 2019**	**July 22nd, 2019**	**July 23rd, 2019**	**July 25th, 2019**	**July 26th, 2019**	**July 28th, 2019**	**July 30th, 2019**	**August 1st, 2019**	**August 2nd, 2019**
Hemoglobin (g/dL)	10.94	10.99	8.6	7.9	8.0	10.1	10.0	11.39	8.11	8.31	7.90
White blood cells ($10^3/mm^3$)	37.930	31.520	18.790	20.000	22.350	29.150	34.620	21.920	13.080	12.310	9.856
Platelets ($10^3/mm^3$)	188.100	196.700	128.400	146.300	120.700	218.300	321.000	537.000	367.400	235.100	238.400
Creatinine (mg/dL)	3.7	4.2	3.1	3.7	3.0	4.8	5.3	4.2	3.8	2.1	1.8
Alkaline phosphatase (IU/L)	378	...	...	221	...	...	...	339	...	...	...
Amylase	499	...	...	...	...	...	...	...	...	...	...
Gamma-glutamyl transferase (IU/L)	...	...	...	...	...	...	...	73	...	...	...
Glucose (mg/dL)	128	...	...	119	...	...	86	86	...	...	...
Potassium (mmol/L)	5.0	5.3	3.9	3.7	3.5	3.6	3.9	4.0	3.5	4.2	3.8
Sodium (mmol/L)	135	132	140	142	140	137	135	137	139	138	139
Calcium	...	...	8.4	8.2	8.7	9.4	10.3	10.0	8.9	9.0	8.4
Magnesium	...	2.2	2.8	2.5	2.2	2.6	2.4	2.3	1.7	...	...
Aspartate aminotransferase (IU/L)	4009	4979	1884	976	435	181	97	91	41	36	36
Alanine aminotransferase (IU/L)	590	818	856	644	520	297	233	187	124	92	92
Urea (mg/dL)	162	188	137	131	130	176	135	86	104	97	96
Albumin (g/dL)	3.0	3.2	3.5	3.4	3.4	3.1	3.3	3.8	3.4	...	...
Creatine phosphokinase (IU/L)	...	407.708	99.986	88.874	21.006	6.655	...	882	306	...	208
Creatine phosphokinase -MB (IU/L)	...	3.888	1.385	691	288	133	...	48	26	...	30
Lactate dehydrogenase (IU/L)	12.753	...	9.803	...	5.658	4.296	...	3.275	1.726	...	...

Reference values: Hemoglobin: 13.0-16.0 g/dL for males; White blood cells: 4.000-10.000/mm³; Platelets: 130,000-400.000/mm³; Creatinine: 0.3-1.0 mg/dL for children; Alkaline phosphatase: male ≤390 IU/L; Gamma-glutamyl transferase: 7-60 IU/L for males; Glucose: <99 mg/dL; Potassium: 3.6-5.2 mmol/L; Sodium: 135-145 mmol/L; Aspartate aminotransferase: 2-38 IU/L; Alanine aminotransferase: 2-44 IU/L; Urea: 10-45 mg/dL; Albumin: 3.5-5 g/dL; Creatine phosphokinase: 24-190 IU/L; Creatine phosphokinase-MB: 2-25 IU/L; Lactate dehydrogenase: 211-423 IU/L; pH: 7.35-7.45; pCO_2: 35-45; pO_2: 80-100; sO_2: 94-97%; Lactate ≥45 mg/dL; Base excess -2 to +2 (mmol/L); HCO_3^-: 22-26 mmol/L.
