# Supplementary material for: Bee sting envenomation severe cases in Manaus, Brazilian Amazon: clinical characteristics and immune markers of case reports
Source: Rev Soc Bras Med Trop. 2020 Dec 21;54:e20200319. doi: 10.1590/0037-8682-0319-2021 (PMC7747811; doi:10.1590/0037-8682-0319-2021)
Supplement: Supplementary file 3 [file 1678-9849-rsbmt-54-e20200319-suppl3.pdf]

Supplementary material: Material and Methods

Biological sample collection

Approximately 4 mL of peripheral blood was collected at admission from each patient and from 20 age-matched healthy controls by venous puncture in tubes containing EDTA (BD Vacutainer® EDTA K2). Samples were centrifuged (3.000 rpm, 10 min) and plasma aliquots were stored in a freezer at -80°C for measurement of inflammatory molecules.

Immune markers measurements

The plasma soluble immune markers (CXCL-8, CCL-5, CXCL-9, CCL-2, CXCL-10, IL-6, TNF, IL-2, IFN-γ, IL-17A, IL-4 and IL-10,) were quantified using the Cytometric Bead Array (CBA). BD™ Human Chemokine Kit (BD Biosciences, San Diego, CA, USA) and BD™ Human Th1, Th2, Th17 Cytokine Kit (BD Biosciences, San Diego, CA, USA) were used following the manufacturer's technical guidelines and protocols. A FACS Canto II flow cytometer (BD Biosciences, San Jose, CA, USA) at Fundação Hospitalar de Hematologia e Hemoterapia do Amazonas (HEMOAM) was used for sample acquisition. FCAP-Array software v3 (Soft Flow Inc., USA) was used to calculate the cytokine levels (MFI). The analyses were performed in triplicate.

CNAs measurements

CNAs were isolated from plasma from patients or healthy donors using the QIAamp DNA Blood Mini Kit (QIAGEN, Chatsworth, CA, USA) according to the manufacturer's instructions. To quantify CNAs levels in plasma, amplification of the genomic sequence of the human telomerase reverse transcriptase (hTERT), a ubiquitous single copy gene mapped on 5p 15.33, was used as a marker of the total amount of DNA present in the samples. We used the following specific primers: **F:** *5GGC ACA CGT GGC TTT TCG 3*; **R:** *5 GGT GAA CCT GCT AAG TTT ATG CAA 3*; **Probe VIC5:** TCA GGA CGT CGA GTG GAC ACG GTG-3 TAMRA, as previously described (1). The DNA was amplified using an Applied Biosystems 7500 Fast System®. To normalize the amount of DNA in plasma samples, we used the dilution of plasmids containing the target region. The analysis was performed in triplicate.

Data analysis

Data analyses were performed using the software GraphPad Prism (v5.0). The cases' results were compared to the control group, which consisted of 20 healthy subjects, with ages ranging from 22 to 36 years (median = 27 years), all living in Manaus-AM. The values for each inflammatory molecule were compared with the interquartile range (IQR) values of the reference group.

References

1. Sozzi G, Conte D, Leon M, Ciricione R, Roz L, Ratcliffe C, et al. Quantification of free circulating DNA as a diagnostic marker in lung cancer. J Clin Oncol. 2003;21(21):3902–8.
